# Deep learning approaches for classification tasks in medical X-ray, MRI, and ultrasound images: a scoping review

**DOI:** 10.1186/s12880-025-01701-5

**Published:** 2025-05-07

**Authors:** Hafsa Laçi, Kozeta Sevrani, Sarfraz Iqbal

**Affiliations:** 1 Department of Statistics and Applied Informatics, Faculty of Economy, University of Tirana, Tirana, Albania; 2 Department of Informatics, Faculty of Technology, Linnaeus University, Växjö, Sweden

**Keywords:** Deep learning, Medical image classification, MRI, Ultrasound, X-ray

## Abstract

Medical images occupy the largest part of the existing medical information and dealing with them is challenging not only in terms of management but also in terms of interpretation and analysis. Hence, analyzing, understanding, and classifying them, becomes a very expensive and time-consuming task, especially if performed manually. Deep learning is considered a good solution for image classification, segmentation, and transfer learning tasks since it offers a large number of algorithms to solve such complex problems. PRISMA-ScR guidelines have been followed to conduct the scoping review with the aim of exploring how deep learning is being used to classify a broad spectrum of diseases diagnosed using an X-ray, MRI, or Ultrasound image modality.

Findings contribute to the existing research by outlining the characteristics of the adopted datasets and the preprocessing or augmentation techniques applied to them. The authors summarized all relevant studies based on the deep learning models used and the accuracy achieved for classification. Whenever possible, they included details about the hardware and software configurations, as well as the architectural components of the models employed. Moreover, the models that achieved the highest accuracy in disease classification were highlighted, along with their strengths. The authors also discussed the limitations of the current approaches and proposed future directions for medical image classification.

## Introduction

Deep learning has provided accurate solutions in the healthcare system [1], and the future of deep models seems to be very promising [2]. However, the development of robust deep learning models requires a lot of effort and faces so many challenges related to the employed dataset characteristics, image preprocessing techniques adopted, and the technical setups or the architectural components of the implemented models [3]. Computational efficiency is another challenge because deep models require considerable memory capacity to reach state-of-the-art performances on available datasets [4].

For a better understanding of the deep learning approaches used to perform medical image classification tasks, a scoping review is conducted in this study. Authors have taken into consideration only X-ray, MRI, and Ultrasound image modalities due to their wide clinical application, versatility, accessibility in medical institutions, and cost-effectiveness [5, 6]. Although there are several related reviews in the literature, they vary in scope and coverage. For example, reviews in [7–14] cover specific conditions such as diabetic retinopathy [7], rare diseases [8], breast cancer [9], covid-19 [10, 11], musculoskeletal malignancies [12], skin cancer [13], and psychiatric diseases [14], respectively. Similarly, the authors in [15] explore the use of deep learning in dental regeneration and rehabilitation procedures with a focus on segmentation and object detection tasks. Other reviews are concentrated on transfer learning tasks applied to neuroimaging [16] and other modalities [17]. Moreover, there is a complete review presented by the authors in [18] on medical image preprocessing, but it has a strict focus on image-denoising techniques. They performed an in-depth analysis of noise sources and performance indicators; however, numerous other image preprocessing and augmentation techniques are adopted by the state-of-the-art to improve the quality of the input datasets for their deep learning models.

In contrast to previous work, this review focuses specifically on image classification tasks and has a broader scope, meaning that it is not limited to specific conditions, but instead spans across multiple diseases. It also provides details about the size and accessibility of the datasets used in existing experimental studies to offer researchers practical insights for selecting appropriate datasets or assessing the reproducibility of results. In addition, the authors highlight the preprocessing and augmentation techniques employed in image classification studies. Through that researchers can understand which techniques worked best and which ones were overlooked or need further exploration. Furthermore, other reviews tend to remain focused on the algorithms or implemented deep models, often neglecting hardware and software configurations used for implementation. To the best of the authors’ knowledge, there are no review studies that provide details regarding such technical characteristics for each image classification model. By documenting such information in this work the authors help users evaluate whether they have enough resources to replicate the setup in their specific context or if they need to scale up.

This research work is guided by the following questions:

- **RQ1**: What are the most common diseases covered by the studies and what anatomical site/organs do they affect?
- **RQ2**: How are medical image modalities distributed in the selected studies, considering only the sample size and accessibility of the datasets adopted?
- **RQ3**: What are the most applied image preprocessing techniques during the data preparation stage? Is data augmentation required?
- **RQ4**: What are the architectural components and hardware configurations of the models employed by the studies and what deep learning frameworks/libraries are used for their implementation?
- **RQ5**: What are the limitations of the deep learning approaches for image classification?

After applying the inclusion and exclusion criteria described in the methodology, this review considered 80 studies published between 2014 and 2024 for final analysis. The most frequently studied diseases were the ones affecting the lungs, brain, and mammary glands. X-ray image modality was the most commonly used compared to MRI and Ultrasound. In 50% of cases, image classification was conducted on a dataset size ranging between 1 K and 10 K samples. With the increase in the sample size, the adoption of private datasets decreased compared to public ones. 54% of the studies adopted both, preprocessing and augmentation as a pre-training step for the model. The most applied preprocessing techniques were image normalization, image resizing, gray-scaling, and denoising. For augmentation, image rotation, horizontal/vertical flipping, and zooming were the most adopted approaches. The most employed deep learning model was custom CNN and the environment used in the majority of studies for model implementation was Tensorflow as a backend combined with Keras or Google Colab as interfaces. Regarding the architectural components, 54% of the studies used ReLU or LeakyReLU activation functions in the hidden layers and Softmax in the last output layer. Adam optimizer was used in 46% of cases, followed by SGD optimizer in 18%. The majority of the models employing the Adam optimizer performed training using 16GB-64GB of dedicated GPU. Meanwhile, the ones employing SGD optimizer performed training using 16GB of dedicated GPU or less. In Table 4 we present the models with the highest accuracy and for the most studied organs EfficientNet (combined with XAI techniques) and custom CNN both demonstrated great results. Limitations and future directions of authors in this area are mentioned in RQ5. Small dataset size, imbalanced datasets, lack of historical patient information, considering only one image modality per disease, performing binary classification instead of multi-class, incorrect data annotation, and limited hardware capacities were some of the issues affecting the interpretability and generalizability of deep learning models. To address that, the authors suggest applying several augmentation approaches, such as using SMOTE, DARI, and cGAN models, and using pre-trained models. Also, leveraging the benefits of Explainable AI (XAI) techniques can significantly reduce the complexity of the model making it more interpretable.

The rest of the article has been arranged as follows: Sect. “Literature review” portrays brief results from the literature review. Section “Methodology” explains the methodology used in this study. Section “Results & discussions” presents the results and discussion. Section “Open issues” concludes the article with further research suggestions.

## Literature review

Medical imaging plays an important role in different clinical procedures and in the detection or diagnosis of various pathologies [19, 20]. Imaging techniques can easily reach the internal structures of the body and identify a lot of abnormalities [5]. In order to achieve an effective treatment of these abnormalities, image classification needs to be accurate, but this process is usually tedious and prone to errors because of a subjective interpretation by medical experts [21]. Recently, the automation of the disease diagnosis process came with a lot of advantages and potential [22]. An automated solution that can be taken into consideration is machine learning, but medical images are complex in nature compared to other types of images due to data variation from patient to patient, so traditional machine learning is not sufficient [2]. Given that, deep learning makes a good alternative and many review studies about its application in processing medical image data have been published.

Khosravi et al. [23] performed a scoping review on the application of machine learning and deep learning in cardiothoracic imaging. Kim et al. [15] focused their research on the task of transfer learning by providing guidance on how to choose a backbone CNN model and the appropriate transfer learning approach, in order to correctly perform image classification. Mohammad-Rahimi et al. [8] discussed in their work how deep learning is performed on periodontal or oral implantology tasks using classification, segmentation, and object detection techniques. Lee et al. [7] explored how deep learning is advancing in rare disease diagnosis, including rare neoplastic diseases, rare genetic diseases, and rare neurological diseases.

Tsiknakis et al. [9] focused on diabetic retinopathy segmentation, classification, and detection through deep learning, using fundus images. Mao et al. [10] performed a systematic scoping review to summarize the contribution of machine learning and deep learning in the classification of breast tumor using ultrasound elastography. Gillman et al. [11] and Wang & Hargreaves [16] focused their research on the classification of COVID-19 from chest radiological images and reviewed deep learning techniques adopted in that direction.

Another scoping review about transfer learning approaches is conducted by Ardalan & Subbian [17], but this time about neuroimaging analysis. Meanwhile, Morid et al. [12] explored the use of transfer learning on medical image analysis using ImageNet. Deep learning on musculoskeletal malignancy diagnosis was studied by Hinterwimmer et al. [13] and the article had an explorative nature. More scoping reviews are conducted for skin cancer detection [14] and human brain neurological and psychiatric diseases [24].

## Methodology

**Fig. 1:**
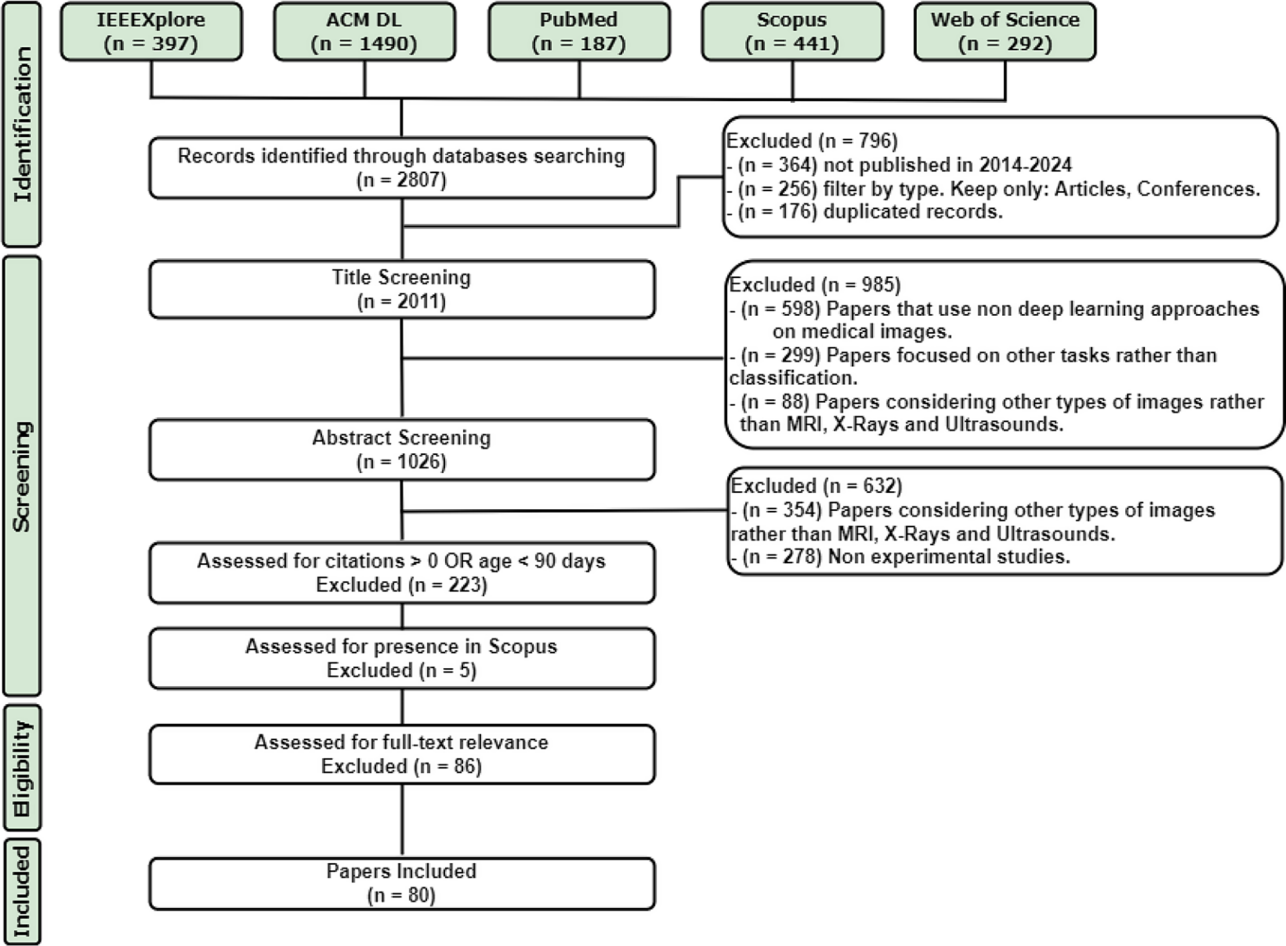
Flow of the study selection process

A scoping review was conducted following the PRISMA-ScR checklist [25] and the updated methodological guidance [26]. Figure 1 shows the step-by-step procedure needed for the systematic selection and screening of the available studies in the research area.

### Selected databases and search strategy

**Fig. 2:**
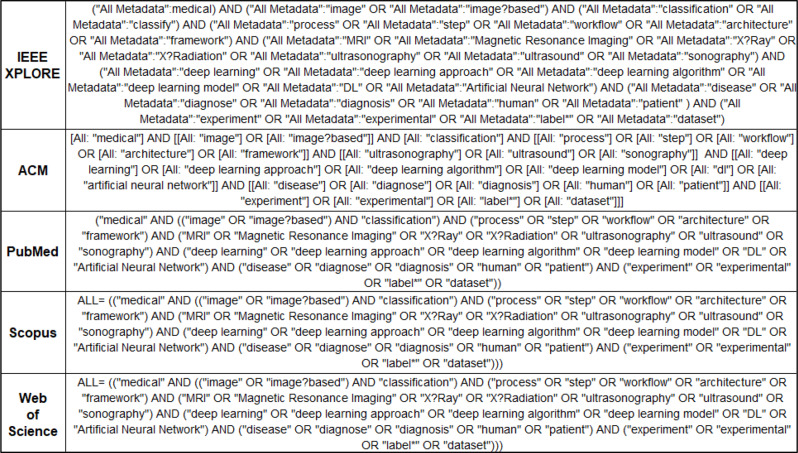
Search queries performed in the chosen databases

The five well-known digital libraries that were chosen to perform the research and identify the relevant studies are mentioned below along with the respective search queries applied as shown in Fig. 2. Their combination covered a wide variety of papers about the application of technology in medicine.

### Selection criteria

The total number of papers gathered from the electronic search during the identification stage was 2807. These studies were filtered considering the publication period, publication type, and presence of duplicates. The remaining number of papers (2011) underwent a screening process by titles, abstracts, and other exclusion criteria, reducing in this way the number of papers to 166. The latter were assessed for full-text relevance and finally, 80 papers were found eligible for this scoping review. Table 1 shows the inclusion and exclusion criteria applied.

**Table 1:** Criteria applied to include or exclude studies for our scoping review

Inclusion Criteria	Exclusion Criteria
Studies published from 2014 to 2024.	Nonexperimental studies or studies that have not solved a specific classification problem.
Journal articles and conference proceedings.	Studies not leveraging a deep learning approach or not adopting a deep learning model as a problem solver.
Studies written in English.	Studies being published for 3 months or more and having 0 citations.
Studies focused only on the classification task and not in other deep learning tasks.	Books, Reviews, Early access articles, etc.
Studies considering only MRI, X-ray, or Ultrasound image modalities to perform classification.	To date Web of Science and Scopus are two of the most important databases, but Scopus provides a wider content coverage [27]. So, studies not present and cited in Scopus were excluded.

## Results & discussions

In the upcoming sections, we will offer a more detailed analysis and interpretation of our findings. We will begin by describing the relevant studies and examining publication trends. Each subsequent section will address one of the five research questions outlined in this scoping review. We will conclude with a discussion of open issues and suggest future trends that researchers should explore.

### Description of the relevant studies

**Fig. 3:**
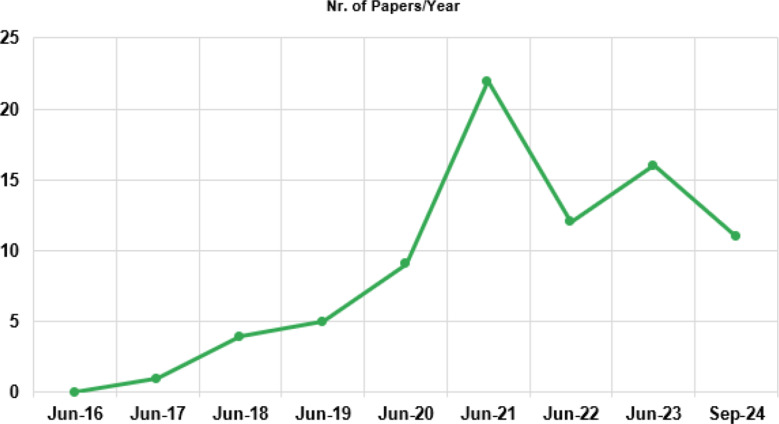
Papers published annually from 2016 to 2024

The number of papers published from 2016 to 2024, following a deep-learning approach for the classification of medical images is displayed in Fig. 3. It helps us to gain a better understanding of the evolution of the subject matter and to provide context for the period in which researchers have been publishing in this area. The graph is limited to the number of studies chosen for the final screening phase of this scoping review and it demonstrates that numbers have risen rapidly over the last seven years. A growing trend of publications is reported starting from the second half of 2017 to the first half of 2020, but a sharp rise is seen in the second half of 2020 with 12 relevant papers, which coincides with the expansion of COVID-19. Even if we can spot a decrease in the second half of 2021, the number of pertinent papers published remains significant. Given that, we can certainly say that this is still a trending topic.

### RQ1: What are the most common diseases covered by the studies and what anatomical site/organs do they affect?

The majority of studies (34%) were focused on pathologies or diseases affecting the lungs, for instance, COVID-19, pneumonia/pediatric pneumonia, and pulmonary tuberculosis. Diseases affecting the brain, including Alzheimer’s, dementia, brain tumors, autism spectrum disorder, and cortical tubers, were also present in a considerable number of studies, 31% to be more precise. Another anatomical site considered in 10% of the articles by researchers was the mammary gland. In the analyzed papers, diseases affecting the thoracic cavity and the organs it includes, liver, thyroid gland, bones, spine, uterus, and prostate gland were the least studied, 8%, 6.25%, 5%, 2.5%, 1.25%, 1.25%, 1.25% respectively. Furthermore, regarding the imaging modality, X-rays were the most frequently used for disease diagnosis (44%), followed by MRI image modality (35%) and Ultrasound image modality (21%). The distribution is displayed in more detail in Table 2.

**Table 2:** Distribution of the classified diseases, anatomical sites affected, and medical image modalities used in the relevant studies

Ref.	Disease	Anatomical Site/Studied Organ	Medical Imaging Modality	Frequency
[28–51]	Covid-19, Pneumonia, Pediatric Pneumonia	Lungs	Posterior-Anterior CXR	30%
[52–66]	Alzheimer Disease, Dementia	Brain	MRI/sMRI/ T1-weighted MRI	19%
[67–72]	14-Thoracic Diseases (Atelectasis, Cardiomegaly, Effusion, Infiltration, Mass, Nodule, Pneumonia, Pneumothorax, Consolidation, Edema, Emphysema, Fibrosis, Pleural Thickening, and Hernia)	Organs of the thoracic cavity	CXR	8%
[73–79]	Brain Tumor, Glioma Tumors	Brain	MRI, T2-SWI MRI	9%
[80–83]	Thyroid Nodules	Thyroid Gland	Ultrasound	5%
[84–91]	Breast Cancer	Mammary Gland	Ultrasound	10%
[92, 93]	Bone Fracture, Femur Fracture	Bones	Bone X-Ray	2.5%
[94–96]	Pulmonary Tuberculosis	Lungs	CXR	3.7%
[97, 98]	Liver lesions/Liver tumor	Liver	DW-MRI, Ultrasound	2.5%
[99, 100]	Liver fibrosis/Hepatic fibrosis	Liver	Ultrasound	2.5%
[101, 102]	Autism Spectrum Disorder (ASD), DOC	Brain	rs-fMRI	2.5%
[103]	Cortical Tubers	Brain	MRI	1.25%
[104]	Disc Herniation	Spine	Axial MRI	1.25%
[105]	Fetal Malposition	Uterus	T2-weighted 3D fetal MRI	1.25%
[106]	Voxel-Level Liver Stiffness	Liver	MRE (MRI + low-frequency vibrations)	1.25%
[107]	Prostate Cancer	Prostate Gland	Ultrasound	1.25%

As a final point, 77% of the studies using X-ray imaging modality are used to classify diseases affecting the lungs, and 89% of the studies using MRI imaging modality are used to classify diseases affecting the brain. Ultrasound imaging modality is mostly used to classify diseases affecting the mammary gland (47% of the studies).

### RQ2: How are medical image modalities distributed in the selected studies, considering only the sample size and accessibility of the datasets adopted?

Classifying medical images through deep learning methods is considered to be an arduous task because of two main reasons: the insufficient amount of medical data available to train the models and the absence of medical specialists needed for the data labeling process [108]. As a matter of fact, the lack of data is a limitation in any field of study, but what makes it more challenging for medical images are data privacy issues [109]. Consequently, a large number of studies consider publicly available datasets to perform classification.

**Fig. 4:**
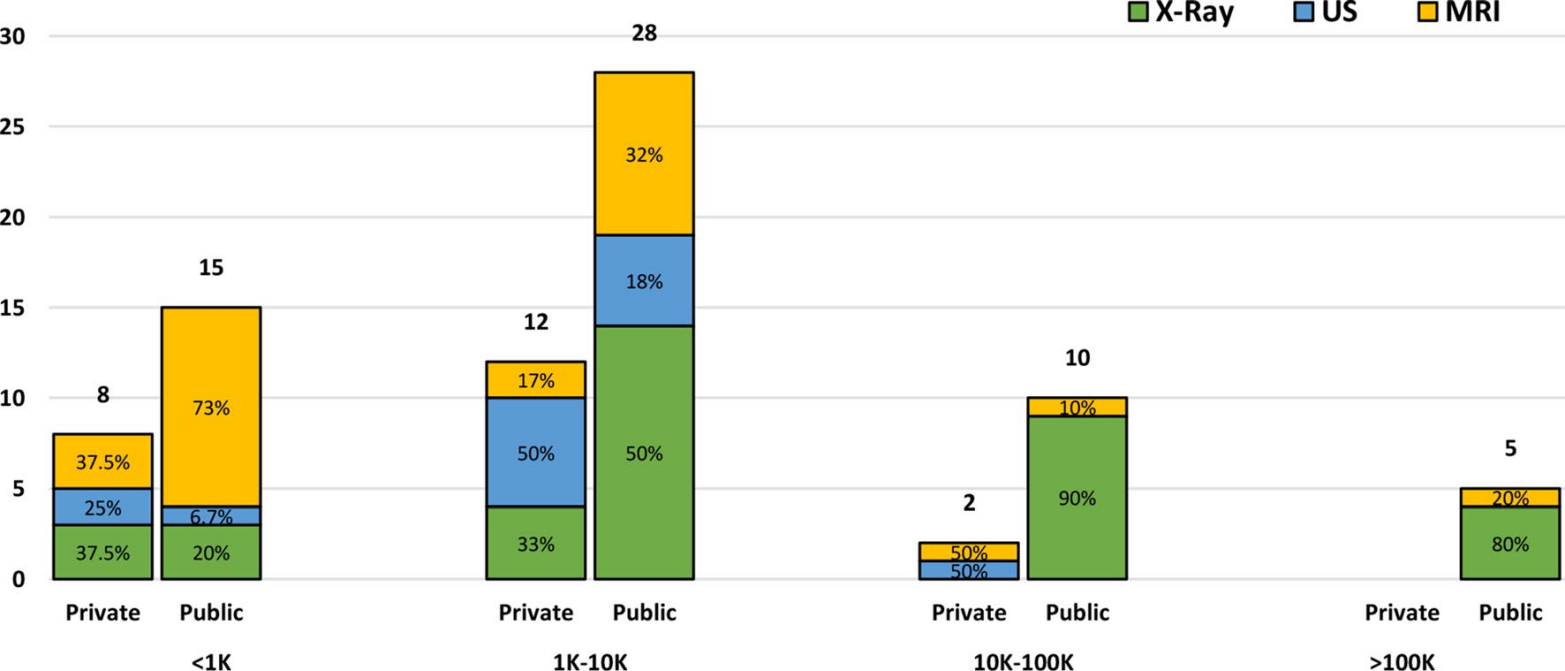
Frequency of studies using a specific medical imaging modality for different dataset sizes and accessibility

Through this section, the authors aim to understand the availability of data for various diseases, to get insights into the trends of public or private data-sharing practices, and to emphasize collaborative initiatives between researchers. The sample size and accessibility of the datasets adopted by the studies considered in our research are shown in Fig. 4. The largest number of studies (50%) fall in the second category, where the image classification task is conducted with a dataset size starting from 1 K to 10 K samples. The number of studies decreases for datasets with more than 10 K samples and the category with the least number of studies (6.25%) is the last one, which performs image classification with a sample size greater than 100 K. As for accessibility, public datasets have expectedly the highest frequency for each category (65%, 70%, 83% and 100%). Notice the fact that with the increase in the sample size, decreases the adoption of private datasets compared to public ones.

Figure 4 gives information about the distribution of imaging modalities as well. In the first category (< 1 K), We can observe an equal distribution (37.5%) between the X-ray and MRI samples in the private datasets. 73% of the public datasets comprise MRI samples. In the second category (1–10 K), 50% of the private datasets comprise Ultrasound images and 50% of the public datasets comprise X-ray images. For the last two categories, 90% and 80% of the public datasets, respectively, consist of X-rays. As a final observation, it is noted that the majority of datasets composed of Ultrasound images are private, while datasets composed of X-ray or MRI images are mostly public.

Additionally, among the articles of the first category (< 1 K), the ones performing the image classification task for diseases affecting the brain occupy 50% of the studies. In the second and third categories, the majority of articles classify diseases affecting the lungs (40% and 67%). In the last category (> 100 K) the articles mostly classify diseases affecting the thoracic cavity.

### RQ3: What are the most applied image preprocessing techniques during the data Preparation stage? Is data augmentation required?

When it comes to classification problems the quality of the input has revealed to be crucial, especially while using deep learning approaches. Different preprocessing techniques are being applied to raw data before feeding them to the deep models in order to improve their quality [110].

**Fig. 5:**
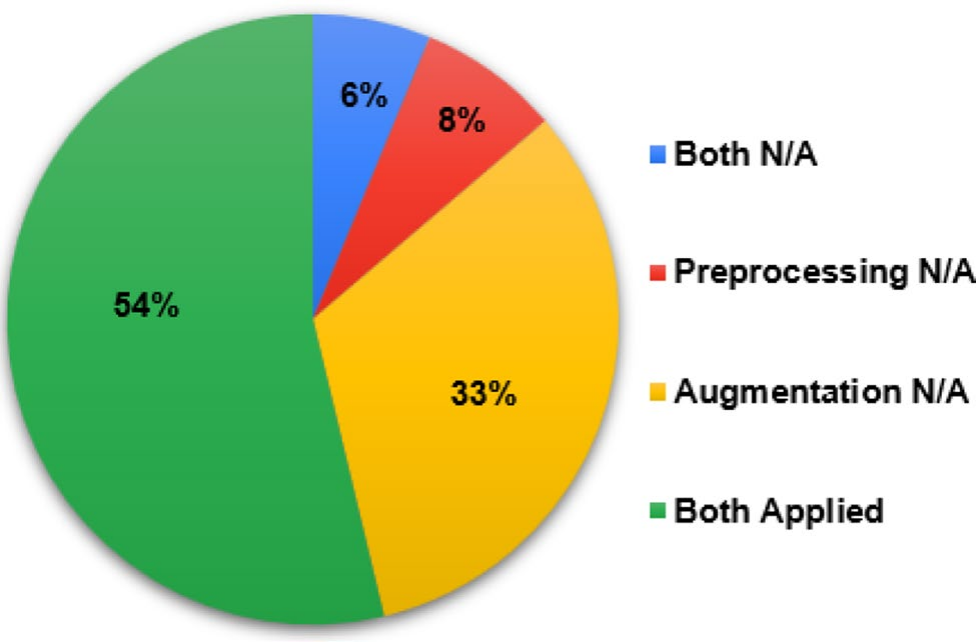
Adoption of preprocessing & augmentation by the eligible studies

Figure 5 shows that over half of the studies (54%) adopted both, preprocessing, and augmentation as a model pre-training step. Only 26 studies (33%) have not applied (N/A) data augmentation, hence the adoption of the latter appears to be important.

**Fig. 6:**
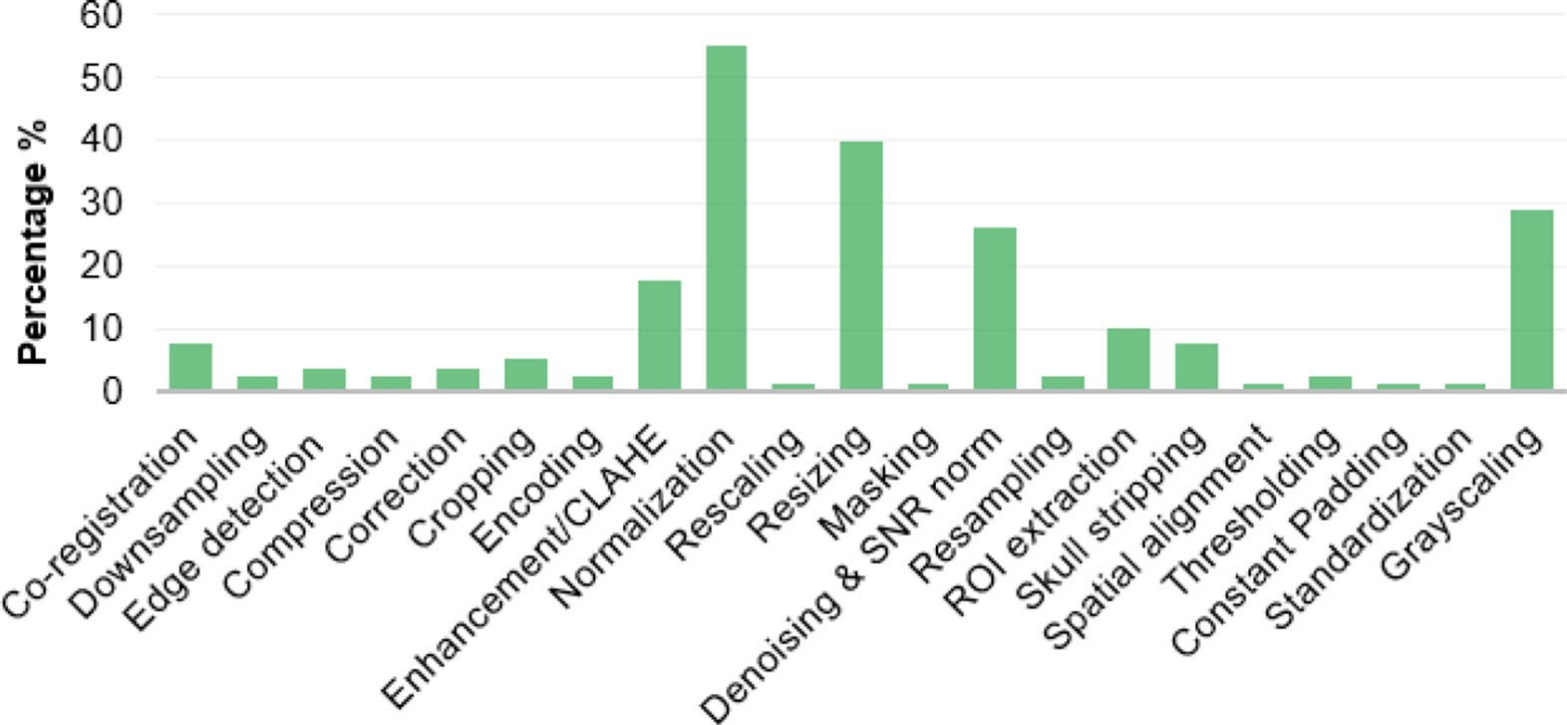
Percentage of papers using a specific preprocessing technique

Figure 6 shows the frequency of studies using specific preprocessing techniques. Image normalization, image resizing, gray-scaling, and denoising were the most frequently applied, in 55%, 40%, 28%, and 26% of the papers respectively, followed by image enhancement (18%). For image denoising, an effective approach to be considered is the integration of.

**Fig. 7:**
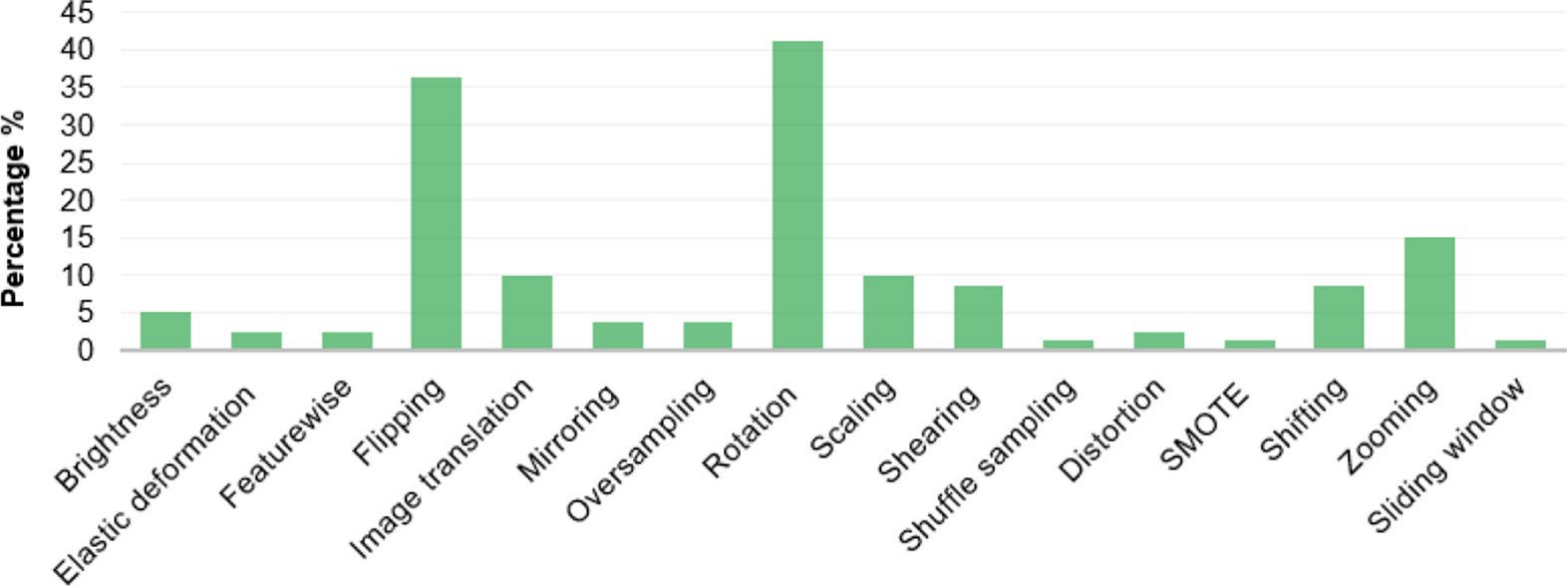
Percentage of papers using a specific augmentation approach

The frequency of studies using specific augmentation approaches is displayed in Fig. 7. Image rotation (41%), horizontal/vertical flipping (36%), and zooming (15%) were the most used methods to increase the dataset size. Scaling (10%), image translation (10%), shifting (9%), and shearing (9%) seem to be significant as well.

### RQ4: What are the architectural components and hardware configurations of the models employed by the studies and what deep learning frameworks/libraries are used for their implementation?

Table 3 organizes the relevant studies according to the deep learning model employed, the dataset size, and the accuracy achieved during training. It provides also details regarding the hardware and software configurations adopted for implementation. Whenever there was evidence, we extracted the time taken by the model for training. The majority (36%) employed custom CNNs, and a vast part (21%) utilized a hybrid approach to classify images. Several other models, including ResNet (9%), VGG-16 (7.5%), EfficientNet (6.25%), DenseNet (5%), and Genetic DCNN (3%) were used as well. The remaining part adopted RNN, DNN, AlexNet, Self-Attention Transformer, DeepLabV31, Attention-based CNN, IEViT, ANN, InceptionV3, and FCN as a solution for the classification task.

**Table 3:** Classification based on the deep learning model adopted, accuracy achieved and technical implementation details

Ref	Disease	Dataset Size	Model	Acc.%	Technology/Environment
[28]	Pneumothorax disease	Dataset1: 12,047 Dataset2: 3315	Combines 3 CNN: VGG-16, VGG-19, DenseNet-121	82.68	• SW: Keras + TensorFlow + Python
[97]	Liver lesions/Liver tumor	130	CNN	83	• SW: TensorFlow + Python
[29]	Covid-19	5000	genetic DCNN	98.84	• SW: TensorFlow • HW: NVIDIA Tesla TitanXp GPU, 512 GB memory, 240 SSD Intel i7 2.50 GHz
[52]	AD and Dementia	6400	CNN	95.23	• HW: NVIDIA Quadra RTX6000 workstation (24GB GPU)
[94]	Pulmonary Tuberculosis	662	VGG16	93.18	Not Mentioned
[30]	Covid-19	82,670	CNN	91.53	• SW: PyTorch implementation (https://github.com/IliasPap/COVIDNet)
[31]	Covid-19	33,231	DARI/GAN + CNN	93.94	• SW: Keras + TensorFlow library + Python’s Matplotlib library. • HW: Nvidia RTX 2060 GPU, Intel Core i7 workstation, 3.00 GHz CPU, 16 GB RAM
[32]	Covid-19	5863	ResNet-50 + MLP	91	Not Mentioned
[53]	AD	427	RNN	86	• SW: PRO version of Google Colab cloud service + Keras
[67]	Thoracic diseases	112,120	CNN	-	Not Mentioned
[33]	Covid-19	2905	cGAN + U-Net + ResNet-50	97.8	• SW: Tensorflow API on Google Colab Pro Cloud platform.
[34]	Covid-19	5956	CNN	89.47	• SW: python version 3.7.3 + Keras + TensorFlow2.0.0 • HW: NVIDIA Quadro k620 GPU with 2GB GPU, Intel Corei7-3.6 GHz
[35]	Pneumonia	5856	VGG16	97.4	• SW: Keras v2.2.5 + TensorFlow v1.14.0 • HW: Foxconn HPC M100-NHI with an 8-GPU cluster of NVIDIA Tesla V100 16GB cards
[68]	Thoracic diseases	108,948	CNN	-	• SW: Caffe framework + Dev-Box linux server with 4 Titan X GPUs
[36]	Covid-19	3000	CNN	97.33	• SW: Keras + TensorFlow 2.0 + Python • HW: NVIDIA Tesla P100 PCIe GPU of 16 GB on Google Colaboratory Server
[73]	Brain Tumor	2406	VGG16	98.4	• HW: NVIDIA Quadro P5000 GPU card with 16GB memory. • Training time: 16 h
[54]	AD	400	CNN	73.4	• SW: Keras + TensorFlow backend • HW: NVIDIA RTX2080 GPU
[37]	Covid-19	1415	CNN	98.62	• SW: Keras + Tensorflow + Python • HW: NVIDIA GEFORCE GTX 1050 Ti 8 GB & 4 GB RAM, Intel corei7 GHz
[55]	AD	4786	CNN	93.21	• SW: Pytorch library • HW: NVIDIA TITAN RTX GPU • Training time: 20 h
[95]	Tuberculosis	4701	AlexNet and GoogleNet	85.68	• SW: Pycaffe + Scikit-learn + Pandas-ml • HW: NVIDIA Tesla K80 GPU • Training time: less than 1 min per image (2 to 3 days training)
[38]	Covid-19	17,990	DenseNet	94.3	Not Mentioned
[104]	Disc Herniation	2329	AlexNet	87.75	• SW: Caffe framework + Ubuntu Linux OS + CUDA Math Library • HW: Intel core i5 6500 (3 MHz) 4GB RAM, a 960 GPU GTX graphics card
[56]	AD	530	CNN	88.31	• SW: Keras + Tensorflow + Python 3.6.6 + Ubuntu16.04-x64/ • HW: NVIDIA GeForce GTX TITAN X
[57]	AD	428	RNN + MLP	89.69	• HW: NVIDIA GeForce GTX 1080 GPU 8GB • SW: Keras + Theano
[39]	Pneumonia	5856	VGG16	96	• SW: Keras + TensorFlow
[58]	AD	6400	CNN	50	Not Mentioned
[59]	AD	56	CNN	97.75	• SW: OpenCV python and OpenCV3
[60]	AD	388	Self-Attention Transformer	88.2	• SW: PyTorch • HW: 4 NVIDIA 2080 TI GPUs
[69]	Thoracic diseases	1000	DNN	-	• HW: Model parallelized in two NVIDIA Tesla V100 graphic cards
[70]	Thoracic diseases	141,400	DenseNet-169 + ResNet-152	-	• SW: PyTorch • HW: NVIDIA GTX Titan V 12GB GPU
[74]	Brain Tumor	1992	CNN	95.78	• SW: Google Colab + PyTorch + Darknet framework • HW: NVIDIA Tesla P100 GPU • Training time: 7 ms per image
[71]	Thoracic diseases	984	DenseNet	99.58	• SW: Keras + Tensorflow
[61]	AD	581	ResNet and Inception	81	Not Mentioned
[40]	Covid-19	12,723	VGG16	99.1	• SW: MATLAB2021b • HW: Personal Workstation 16 GB RAM and 8 GB graphics card
[41]	pneumonia and COVID-19	29,400	DenseNet201 + VGG16 + GoogleNet + MLP	98.19	• SW: Keras + TensorFlow • HW: NVIDIA GEFORCE RTX-3080 Ti 10 GB GPU, Desktop 64GB RAM Intel Corei9-10850 K CPU running at 3.60 GHz
[42]	Covid-19	18,219	CNN	95.57	• SW: Pytorch
[43]	Covid-19	333	CNN + SVM	99.02	• SW: Keras + TensorFlow.
[44]	Covid-19	16,000	DenseNet-201	99.1	• SW: Keras + TensorFlow 2.2.0
[45]	Covid-19	155	CNN	93.5	• SW: Anaconda with Python + TensorFlow • HW: NVIDIA GeForce GTX 1080 8 GB, PC Intel i7 8700 K 3.70 GHz processor, 32 GB DDR4 RAM
[46]	Covid-19, other pneumonia issues	5071	genetic DCNN	97.23	• SW: Tensor flow • HW: NVIDIA Tesla TitanXp GPU, Intel i7 processor 512 GB RAM 240 SSD, and 2.50 GHz
[47]	Covid-19	4573	CNN	98.92	• SW: Windows 10 (64-bit) operating system, Matlab R2019a. • HW: NVIDIA GeForce GTX-850 M GM107 GPU, Intel Core i7 5400 GPU 2.60 Ghz, 16.0 GB RAM. • Training time: Task 1 (1,828 images) 25 min and Task 2 (2,745 images) 49 min
[106]	Voxel-Level Liver Stiffness	149	DeepLabv31	78	• SW: PyTorch • HW: The Pittsburgh Supercomputing Center using NVIDIA V100 16-GB GPU • Training time: 3–5 h
[75]	Brain Tumor	25,000	CNN	99.25	• SW: Keras + Tensorflow • HW: ThinkStation P620 Tower Workstation, NVIDIA Quadro^®^ P2200 16 GB, Lenovo
[92]	Femur Fracture	1347	ResNet-50 + AlexNet	90	• SW: Linux-based workstation with 16 GB RAM, Intel(R) Xeon(R) CPU @ 3.50 GHz and 64 GB GeForce GTX 1080 graphics card
[103]	Cortical Tubers	6318	TSCCNN + InceptionV3 + ResNet50	95	• SW: Keras + TensorFlow + Python 3.6
[48]	Pediatric Pneumonia	6480	ResNet-50 + Xception + MobileNet	95.83	• SW: Keras + Python + Ubuntu 14.04 OS • HW: 64 GB RAM workstation and NVIDIA 1080 Ti graphics card
[101]	Autism Spectrum Disorder (ASD)	459	CNN	70.45	• SW: Keras + Theano + python • HW: PC Intel Core i7 CPU (2.2 GHz) and 16 GB DDR3 memory.
[93]	Bone Fracture	34,000	Hybrid SFNet + Canny + Grey	99.12	• SW: Tensorflow + Python 3.6 + window 10 OS • HW: TITAN X GPU NVIDIA GeForce GTX 128GB RAM, 8 GB dual graphics card
[76]	Glioma Tumors	572	CNN	97	• SW: Keras + TensorFlow + Google Colab
[105]	Fetal Malposition	144	CNN	97.68	• SW: Keras + TensorFlow + Google Colab • HW: NVIDIA-T4 and NVIDIA-P100 GPU
[80]	Thyroid Nodules	1874	ResNet	88.3	• SW: Pytorch framework + Python 3.7 + Ubuntu18.04 OS • HW: 11G 2080Ti GPU, InyterCore i7-6700, 16 GB memory,
[84]	Breast Cancer	1192	BI-RADS-Net (VGG-16)	88.9	Not Mentioned
[81]	Thyroid Nodules	17,447	ResNet50	83	• HW: PC NVIDIA GTX 1080Ti
[99]	Liver Fibrosis	6323	ResNet	85.9	Not Mentioned
[100]	Hepatic Fibrosis	230	CNN	94.29	• SW: Pytorch 1.11.0 • HW: NVIDIA Quadro P400 GPU, and 16 GB RAM, IntelXeon W-2104 CPU @ 3.20 GHz
[82]	Thyroid Nodules	4554	ResNet50	90.6	• SW: Pytorch framework • HW: Geforce GTX 1080 Ti GPU
[85]	Breast Cancer	1400	VGG16 + ResNet34 + GoogLeNet	91 80 90	• SW: Python 3.6.9 + PyTorch 1.1.0. • HW: Workstation NVIDIA TITAN XP 12 GB, Intel i9 CPU, and 48 GB main memory
[83]	Thyroid Nodules	508	ResNet18	98.4	Not Mentioned
[86]	Breast Cancer	1052	Fus2Net- a novel CNN	92	• SW: Keras + TensorFlow2.0 + Python + Windows 10 • HW: NVIDIA 1080Ti 16GB with CUDA 3584 cores GPU, Intel Corei7-8700k CPU 3.70 GHz, 16.0GB RAM
[98]	liver tumors	2168	USC-Enet (based on EfcientNet-B0)	95.6	• SW: Pytorch + CUDA • HW: 32GB RAM NVIDIA Quadro P600 with 8G video memory, Intel Core i7-8850 H CPU 6cores and 12 threads, core frequency 2.60 GHz
[87]	Breast Cancer	1030	DeepBraestCancerNet (custom CNN)	99.35	• SW: MATLAB R2020a • HW: Intel Corei5-5200U processor and 8 GB of RAM
[102]	disorders of consciousness (DOC)	151	3D EfficientNet-B3	92.7	• HW: NVIDIA Tesla V100 32GB Volta GPU
[49]	Pneumonia	5856	EfficientNetV2L	94.02	• SW: TensorFlow framework
[50]	Covid-19, Pneumonia, Pediatric Pneumonia	4523	Resnet	95.97	• SW: PyTorch 2.1.0 + Python3.10.12 + Windows11 Pro OS • HW: Intel^®^Core™i7 10,700 CPU @2.90 GHz and 32GB RAM
[72]	thoracic disease	312,120	Attention-based CNN	84.67	Not Mentioned
[62]	AD	930	DenseNet-169	98.53	• SW: Pytorch + CUDA + Ubuntu 20.04 OS • HW: 4 AMD vCPUs, and 32 GB of RAM with a 16 GB NVIDIA T4 GPU
[51]	tuberculosis, pneumonia, COVID-19	56,334	IEViT	98.59	• SW: Keras + TensorFlow + vit-keras 4 (https://github.com/faustomorales/vit-keras)
[88]	Breast Cancer	1328	ANN	0.9548	Not Mentioned
[96]	Tuberculosis	5600	Efficient Xception CNN	99.29	• SW: Python 3.9 • HW: 8GB NVIDIA Quadro P4000 GPU, Workstation Intel Core i7 8th processor, 16 GB of RAM
[89]	Breast Cancer	10,714	AlexNet + MobileNetV2 + ResNet	97.75	• SW: MATLAB R2020b • HW: NVIDIA GeForce RTXA4000 16GB GPU, and 64GB RAM, Intel Xeon Core i7 processor, 3.50 GHz
[90]	Breast Cancer	1203	InceptionV3	81	• SW: Keras 2.8.0 + Tensorflowr 2.8.0 • HW: 16 GB of RAM, a GPU-based graphic card with 2176 CUDA cores (GeForce RTX 2060-A8G), and Intel Xeon CPU
[79]	Brain Tumor	351	3D CNN	96.49	• SW: Keras + Tensorflow + Python 3.4 • HW: NVIDIA GPU Geforce GTX 1080 Ti 11GB RAM, Intel-i7 2.60 GHz CPU, 19.5GB RAM
[63]	AD	850,080	ResNet18	97.92	• SW: Caffe + FloydHub cloud service • HW: NVIDIA Tesla K80 GPU
[64]	AD	20,060	3D-CNN	86	• HW: NVIDIA TitanX GPU • Training time: 60 h per image
[107]	prostate cancer	21	CNN	89.9	• HW: Windows system Intel core i5 and 16G memory
[77]	Brain Tumor	2870	CNN + Multi-Branch Network + Inception block	99.3	Not Mentioned
[78]	Brain Tumor	7023	FCN	95	• SW: TensorFlow + Scikit-learn
[65]	AD	6400	CNN	98.63	• HW: 5 GB 421 NVIDIA P2000 GPU, PC Intel Xeon 2687 W v4 (3.0 GHz clock 420 speed, 12 cores, and 24 threads) CPUs, 64 GB RAM https://github.com/shahidzikria/ADD-Net
[66]	AD	1140	3D-CNN	85	Not Mentioned
[91]	Breast Cancer	780	EfficientNet-B7 and Explainable AI	99.14	Not Mentioned

To boost the deep learning community development, plenty of open-source frameworks and libraries are available for the implementation of deep learning models [111]. TensorFlow, Caffe, PyTorch, CNTK, MXNet, Chainer, Theano, and Keras are some of the most popular adopted by the state-of-the-art [112]. In this scoping review, 39% of the considered studies used Tensorflow as a backend combined with Keras or Google Colab as interfaces. PyTorch was mostly used alone for the implementation and training of the models in 18% of the studies, but in some of them, it was combined with Utils, CUDA, and Keras. Caffe was employed alone in 5% of the studies, MATLAB in 5% of the studies too, while Theano as the backend combined with Keras as the interface in 3% of the studies. The remaining part utilized OpenCV or have given details about the hardware, but not regarding the framework or library employed. As shown from the results, TensorFlow is the most frequently used. There are several features that make it a more preferred approach, including fast and distributed computation, effective visualization toolkits, and the ability to support specific hardware configurations [113].

However, choosing the appropriate deep learning environment is not the only challenging task, but also building the model requires effort due to different data characteristics and problems that need to be addressed [114]. In addition to the activation functions and optimizers, there are some parameters like the batch size, number of epochs, and learning rate that should be set manually [115]. These architectural components are all defined in the identified studies of this scoping review and the extracted details can be found in the appendix.

Over half of the studies considered (54%) used mainly ReLU or LeakyReLU activation functions in the hidden layers and Softmax in the last output layer. The rest of the studies applied only ReLU (9%), ReLU with Sigmoid activation function (6%), Sigmoid without ReLU (7.5%), Tanh (1.5%), Softmax with Sigmoid (3%) or have not given details (18%).

As for the optimizers, by making the appropriate choice, researchers can improve weights during training in order to minimize the loss function and enhance the overall performance of the model [78]. 46% of the relevant studies have chosen Adam optimizer, 8% combined Adam with SGD, Adadelta, RMSProp, or Adamax, 18% used SGD, and 5% only RMSProp. A minor part have chosen Nesterov Momentum, Adagrad, ASSOA, Grid Search, and Huddle PSO. For the studies that used Adam and SGD, the other parameters of the models were observed in more detail. This study observed that the largest batch size in the papers using SGD optimizer was 128 [102], while in the papers using Adam optimizer was 400 [37]. Regarding the loss function adopted, cross-entropy was the most used one in both cases.

The hardware configuration is of great importance because it highly affects the training time required by the model [115]. The majority of the models that applied SGD optimizer performed training using 16GB of dedicated GPU or less, while the models that applied Adam optimizer, performed training using 16GB-64GB of dedicated GPU.

Table 4 presents the models that achieved the highest accuracy during classification for each disease studied in the selected papers, along with their strengths. The table indicates that EfficientNet and custom CNN models appear more frequently. Reconnecting to the findings in Table 2, the most considered anatomical sites in the literature were the lungs, brain, and mammary gland. It is important to notice that, regardless of the dataset size and its accessibility, EfficientNet and custom CNN both demonstrate great results when classifying diseases affecting these organs. We want to emphasize that in two cases where EfficientNet results in a high performance, it is combined with XAI, which researchers should consider in their studies.

**Table 4:** The models that achieved the highest accuracy for classifying diseases, along with their strengths

Organ	Disease	Ref.	Model	Acc.%	Strength	Access.	Dataset size	Year
Lungs	Covid-19, Pneumonia, Pediatric Pneumonia	[40]	EfficientNet + VGG16 + ELM + explainable AI	99.1	Computational efficiency of EfficientNet and increased model interpretability through XAI	public	12,723	2022
		[44]	DenseNet-201	99.1	High computational & memory efficiency	public	16,000	2022
	Pulmonary Tuberculosis	[96]	Efficient Xception CNN	99.29	High efficiency + fast training + good generalizability	public	5600	2023
Brain	Alzheimer Disease, Dementia	[65]	CNN	98.63	Synthetic oversampling technique	public	6400	2022
	Brain Tumor, Glioma Tumors	[77]	CNN with Multi-Branch Network + Inception block	99.3	Interpretability and visual explanation of the results through Grad-CAM + Computational efficiency of EfficientNet	public	2870	2023
	ASD, DOC	[102]	3D EfficientNet-B3	92.7	Addressing class imbalance via synthetic oversampling technique	private	151	2024
	Cortical Tubers	[103]	TSCCNN + InceptionV3 + ResNet50	95	Multi-scale extraction of features + efficiency on complex tasks	private	6318	2020
Mammary Gland	Breast Cancer	[87]	DeepBraestCancerNet (Custom CNN)	99.35	customization + flexibility in solution design	public	1030	2023
	Breast Cancer	[91]	EfficientNet-B7 and Explainable AI	99.14	Computational efficiency of EfficientNet and increased model interpretability through XAI	public	780	2024
Thoracic Cavity	Thoracic Diseases	[71]	DenseNet	99.58	High computational & memory efficiency	public	984	2018
Liver	Liver lesions/Liver tumor	[98]	USC-Enet (based on EfcientNet-B0)	95.6	Computational efficiency of EfficientNet and increased model interpretability through XAI	private	2168	2023
Thyroid Gland	Thyroid Nodules	[83]	ResNet18	98.4	Low computational costs	private	508	2022
Bones	Bone Fracture, Femur Fracture	[93]	Hybrid SFNet + Canny + Grey	99.12	Integration of Canny Edge algorithm for feature localization + high efficiency + low computational cost	public	34,000	2022
Spine	Disc Herniation	[104]	AlexNet	87.75	Feature extraction + classification performed in a common structure and the use of real private patient data	private	2329	2019
Uterus	Fetal Malposition	[105]	Fet-Net (CNN)	97.68	Customization + flexibility in solution design	private	144	2023
Prostate Gland	prostate cancer	[107]	CNN	89.9	Customization + flexibility in solution design	private	21	2017

### RQ5: What are the limitations of the deep learning approaches for image classification?

Deep neural networks play an important role in classifying large amounts of complex data accurately and making the correct decisions about them [116]. However, they come along with a lot of challenges that need to be highlighted. In almost all the studies considered for this scoping review, the main limitation mentioned, which directly impacts the deep model performance, is the small size of the data gathered and used for the classification task. This refers to not only private datasets collected from local hospitals but also to publicly available ones. Besides that, the small number of episodes per patient reduces the ability of the model to predict possible future abnormalities. Additionally, a diagnosis does not depend only on what is shown in a medical image modality, but can also be induced from other patients’ information, such as physical conditions, age, gender, lifestyle, etc [71]. These variables are often ignored but considering them as inputs of the model is of great importance.

To detect or classify specific diseases, different image modalities can be used, but in the studies considered the datasets are limited to only one type of image. In addition, images are mostly gathered from a single institution and image parameters differ for different institutions [46, 49, 50, 62, 100, 102]. Moreover, many studies performed binary classification, even if diseases have many categories or stages. Hence, not taking into consideration multiple image modalities, multiple institutions for data collection, and multi-class disease categorization, leads to a lack of the model’s generalizability.

Model evaluation metrics can be impacted by imbalanced datasets, which are the main cause of model overfitting and a very common problem for image classification through deep-learning models [98]. Furthermore, considering the black-box nature of deep learning models and the large number of parameters used for classification, understanding the prediction results becomes a complicated task [117]. Thus, model interpretability has become another challenge identified in the studies. Other issues concerning datasets are the need for medical experts to label the images and the limited hardware capacities to run them [74, 102].

Class imbalance is present in a large number of studies included in this review and it is mainly addressed through preprocessing and data augmentation techniques mentioned in RQ3. In [52] authors have used the Synthetic Minority Over-sampling (SMOTE) technique to balance the dataset by performing a random duplication of minority classes. Another method used by [30, 67, 70, 72] is adding weighted categorical cross-entropy loss function and regularization components in the last dense layers to alleviate the imbalance. In this way, penalties are added during training to shift the focus of the model more to the minority classes. DARI algorithm operates similarly to SMOTE to improve class distribution through oversampling minority classes or undersampling majority ones [31]. An alternative solution considered is the use of the cGAN model [33] to increase the number of images in the underrepresented class through the generator and discriminator networks [68]. have used positive (βP) and negative factors (βN) to adjust the focus of the model equally in the minority and majority classes. An effective approach is the use of a pre-trained model to have a strong feature foundation and then fine-tuning it on the given dataset [95].

It is worth noting that many researchers are leveraging the benefits of Explainable AI (XAI) as a set of techniques that bridges the gap between model complexity and its diagnosis transparency. XAI is becoming crucial in addressing issues such as generalizability, class imbalance, and interpretability. For instance, authors in [118] exploited the Guided Backpropagation XAI technique to improve denoising in the Feature-guided Denoising CNN that they proposed to effectively perform noise removal from portable ultrasound images while preserving important image features. Meanwhile, in [119] they developed a feature-preserving loss function using gradient-based XAI and achieved good results in terms of generalization and interpretability. Also, including segmentation as a prior step to classification has revealed to be important for so many studies due to the impact it has on effectively increasing accuracy [120]. Some of the identified studies of the review, strongly suggest the use of segmentation [28, 37, 73, 97], particularly ‘U’ shaped architectures or fully CNNs [103], attention mechanisms [72, 98] and applying appropriate visualization techniques/tools [53, 61, 98]. However, XAI plays a significant role even during segmentation by ensuring that the model considers relevant anatomical features as the basis for the final image classification. Authors in [121] proposed GradXcepUNet for image segmentation. They combined U-Net with Grad-CAM XAI technique to identify critical regions of medical images resulting in a higher Dice coefficient compared to other state-of-the-art methods.

## Open issues

The scoping review performed showed that diseases affecting the lungs, brain, and mammary glands received the most attention in the relevant studies. This could be due to the public relevance of specific conditions. For instance, the COVID-19 pandemic shifted the research priorities globally creating gaps in studying other diseases [40, 44]. Also, lung, brain, and breast cancer are significant health concerns and early predictions can be life-changing for patients. Other causes for that could be the lack of publicly available datasets or the limited number of specialized clinics. To balance the research directions, other organs and diseases must be targeted. Furthermore, an analysis of effective de-identification or anonymization techniques for medical images before classification can pave the way for future collaborations between researchers and health institutions to increase data accessibility. By strictly following privacy regulations [109] public awareness about the importance of such initiatives can be increased. Moreover, the authors have considered only unstructured data (medical images) to conduct the research, but historical patient data provide important context about diseases and if analyzed, can significantly improve the reliability of results. Besides that, extending the datasets to cover multi-class diseases [29, 52, 59, 105], and considering structured data (personal information, medical history, clinical notes, and patient habits) will bring models closer to real-world scenarios [39, 41, 53, 68]. Also, making the models suitable for classifying other similar pathologies [41, 43, 44, 97, 103] or for more than just one image modality [31, 46, 60], which can be used to diagnose the same pathology, and apply statistical methods to equalize images collected from various institutions [106], would definitely increase the model’s generalizability, and facilitate scaling.

Although this scoping review was limited to experimental studies applied to X-ray, MRI, and Ultrasound, it is necessary to acknowledge that deep learning image classification has been performed on other medical images, as well. Given that, other researchers might extend this work by studying the uncovered modalities. In addition, despite their relevance, this work excludes studies not published in English. Also, it is important to emphasize that we have tried to extract the training time required by the models for the classification tasks performed, but only 9% of the relevant studies had evidence in this regard. Hence, the authors were not able to provide detailed results and discussions on that topic.

## Conclusions

Many systematic and scoping reviews are performed on a specific disease or category of diseases affecting the same organ/anatomical study. In the state of the art, the authors were unable to find a publication that explores and makes a synthesis on the application of deep learning in a broad way. It means covering a wide spectrum of diseases, affecting different organs/anatomical sites of the body, and considering different imaging modalities, especially, with the focus on no other task rather than classification. Therefore, the authors chose to conduct a scoping review which aims to explore and highlight this situation in a better way. By following the PRISMA-ScR guidelines, 80 articles were considered for full-text analysis. The analysis addressed the five research questions outlined in the introduction section.

The findings of this research work emphasize the fact that issues concerning the model’s generalizability, the model’s interpretability, and the size of the imbalanced datasets adopted to train the models are the main limitations present in the state-of-the-art. Possible ways to address such concerns are presented in the limitations section of this review. Furthermore, through the open issues section, the authors outline the gaps that require the attention of the research community, and some future trends.

## Data Availability

The dataset is created by the authors. All the relevant studies used to conduct this scoping review are referenced and the study selection procedure is described in the Manuscript. On reasonable request, the corresponding author can share the calculations performed to answer the research questions.

## References

[1] Lee J, Jun S, Cho Y, Lee H, Kim GB, Seo JB, et al. Deep learning in medical imaging: general overview. Korean J Radiol. 2017;18(4):570–84. 28670152 10.3348/kjr.2017.18.4.570 PMC5447633

[2] Razzak MI, Naz S, Zaib A. Deep Learning for Medical Image Processing: Overview, Challenges and Future. In Conference on Computer Vision and Pattern Recognition; 2018. pp. 323–350.

[3] He Z. Deep Learning in Image Classification: A Survey Report. In 2020 2nd International Conference on Information Technology and Computer Application (ITCA); 2020; Guangzhou, China.

[4] Angelov P, Sperduti A. Challenges in Deep Learning. In The European Symposium on Artificial Neural Networks; 2016; Belgium. pp. 489–496.

[5] Hussain S, Mubeen I, Ullah N, Ud Din Shah SS, Khan BA, Zahoor M et al. Modern Diagnostic Imaging Technique Applications and Risk Factors in the Medical Field: A Review. BioMed Research International. 2022; 2022. 10.1155/2022/5164970 PMC9192206 35707373

[6] Semelka RC, Armao DM, Junior JE, Huda W. Imaging strategies to reduce the risk of Radiationin CT studies, including selective substitutionwith MRI. J Magn Reson IMAGIN. 2007;25(5):900–9. 10.1002/jmri.20895 17457809

[7] Lee J, Liu C, Kim J, Chen Z, Sun Y, Rogers JR et al. Deep learning for rare disease: A scoping review. J Biomed Inform. 2022; 135. 10.1016/j.jbi.2022.104227 36257483

[8] Mohammad-Rahimi H, Motamedian SR, Pirayesh Z, Haiat A, Zahedrozegar S, Mahmoudinia E, et al. Deep learning in periodontology and oral implantology. J Periodontal Res. 2022;57(5):942–51. 35856183 10.1111/jre.13037

[9] Tsiknakis N, Theodoropoulos D, Manikis G, Ktistakis E, Boutsora O, Berto A et al. Deep learning for diabetic retinopathy detection and classification based on. Comput Biol Med. 2021;135. 10.1016/j.compbiomed.2021.104599 34247130

[10] Mao Y, Lim H, Ni M, Yan W, Wong DW, Cheung JC. Breast tumour classification using ultrasound elastography with machine learning: A systematic scoping review. Cancers. 2022;14(2):367. 35053531 10.3390/cancers14020367 PMC8773731

[11] Gillman AG, Lunardo F, Prinable J, Belous G, Nicolson A, Min H, et al. Automated COVID-19 diagnosis and prognosis with medical imaging and who is publishing: a systematic review. Phys Eng Sci Med. 2021;45:13–29. 34919204 10.1007/s13246-021-01093-0 PMC8678975

[12] Morid MA, Borjali A, Del Fiol G. A scoping review of transfer learning research on medical image analysis using imagenet. Comput Biol Med. 2021;128. 10.1016/j.compbiomed.2020.104115 33227578

[13] Hinterwimmer F, Consalvo S, Neumann J, Rueckert D, Eisenhart-Rothe R, Burgkart R. Applications of machine learning for imaging-driven diagnosis of musculoskeletal malignancies—a scoping review. Eur Radiol. 2022;32:7173–84. 35852574 10.1007/s00330-022-08981-3 PMC9474640

[14] Takiddin A, Schneider J, Yang Y, Abd-Alrazaq A, Househ M. Artificial intelligence for skin Cancer detection: scoping review. J Med Internet Res. 2022; 23(11). 10.2196/22934 PMC8663507 34821566

[15] Kim HE, Cosa-Linan A, Santhanam N, Jannesari M, Maros ME, Ganslandt T. Transfer learning for medical image classification: a literature review. BMC Med Imaging. 2022;22(1):69. 35418051 10.1186/s12880-022-00793-7 PMC9007400

[16] Wang Y, Hargreaves CA. A review study of the deep learning techniques used for the classification of chest radiological images for COVID-19 diagnosis. Int J Inform Manage Data Insights. 2022;2(2).

[17] Ardalan Z, Subbian V. Transfer learning approaches for neuroimaging analysis: A scoping review. Front Artif Intell. 2022;5. 10.3389/frai.2022.780405 PMC8899512 35265830

[18] Kaur A, Dong G. A complete review on image denoising techniques for medical images. Neural Process Lett. 2023;55(11):7807–50.

[19] Elangovan A, Jeyaseelan T. Medical imaging modalities: A survey. In 2016 International Conference on Emerging Trends in Engineering, Technology and Science (ICETETS); 2016; Pudukkottai, India.

[20] Cai L, Gao J, Zhao D. A review of the application of deep learning in medical image classification and segmentation. Annals Translational Med. 2020;8(11):713–713. 10.21037/atm.2020.02.44 PMC7327346 32617333

[21] Hebbale S, Marndi A, Manjunatha Kumar BH, Mohan BR, Achyutha PN. A survey on automated medical image classification using deep learning. Int J Health Sci. 2022;6(SP1):7850–65.

[22] Chatterjee P, Dutta SR. A Survey on Techniques used in Medical Imaging Processing. Journal of Physics: Conference Series. 2021;2089(1).

[23] Khosravi B, Rouzrokh P, Faghani S, Moassefi M, Vahdati S, Mahmoudi E, et al. Machine learning and deep learning in cardiothoracic imaging: A scoping review. Diagnostics. 2022;12(10):2512. 36292201 10.3390/diagnostics12102512 PMC9600598

[24] Zhang Z, Li G, Xu Y, Tang X. Application of artificial intelligence in the MRI classification task of human brain neurological and psychiatric diseases: A scoping review. Diagnostics. 2021;11(8). 10.3390/diagnostics11081402 PMC8392727 34441336

[25] Tricco AC, Lillie E, Zarin W, O’Brien KK, Colquhoun H, Levac D, et al. PRISMA extension for scoping reviews (PRISMA-ScR): checklist and explanation. Ann Intern Med. 2018;169(7):467–73. 30178033 10.7326/M18-0850

[26] Peters MDJ, Marnie C, Tricco AC, Pollock D, Munn Z, Alexander L, et al. Updated methodological guidance for the conduct of scoping reviews. JBI Evid Synthesis. 2020;18(10):2119–26. 10.11124/JBIES-20-00167 33038124

[27] Pranckutė R. Web of Science (WoS) and Scopus: The Titans of Bibliographic Information in Today’s Academic World. Publications (MDPI). 2021;9(1).

[28] Iqbal T, Shaukat A, Akram MU, Muzaffar AW, Mustansar Z, Byun YC. A hybrid VDV model for automatic diagnosis of pneumothorax using Class-Imbalanced chest X-Rays dataset. IEEE Access. 2022;10:27670–83.

[29] Babukarthik RG, Adiga VAK, Sambasivam G, Chandramohan D, Amudhavel J. Prediction of COVID-19 using genetic deep learning convolutional neural network (GDCNN). IEEE Access. 2020;8:177647–66. 34786292 10.1109/ACCESS.2020.3025164 PMC8545287

[30] Arias-Londoño JD, Gómez-García JA, Moro-Velázquez L, Godino-Llorente JI. Artificial intelligence applied to chest X-Ray images for the automatic detection of COVID-19. A thoughtful evaluation approach. IEEE Access. 2020;8:226811–27. 34786299 10.1109/ACCESS.2020.3044858 PMC8545248

[31] Sakib S, Tazrin T, Fouda MM, Fadlullah ZM, Guizani M. DL-CRC: deep Learning-Based chest radiograph classification for COVID-19 detection: A novel approach. IEEE Access. 2020;8:171575–89. 34976555 10.1109/ACCESS.2020.3025010 PMC8675549

[32] El-Kenawy ESM, Mirjalili S, Ibrahim A, Alrahmawy M, El-Said M, Zaki RM, et al. Advanced Meta-Heuristics, convolutional neural networks, and feature selectors for efficient COVID-19 X-Ray chest image classification. IEEE Access. 2021;9:36019–37. 34812381 10.1109/ACCESS.2021.3061058 PMC8545230

[33] Liang Z, Huang JX, Li J, Chan S, Enhancing Automated. COVID-19 Chest X-ray Diagnosis by Image-to-Image GAN Translation. In 2020 IEEE International Conference on Bioinformatics and Biomedicine (BIBM); 2020; Seoul, Korea (South). pp. 1068–1071.

[34] Ahmed KM, Eslami T, Saeed F, Amini MH. DeepCOVIDNet: Deep Convolutional Neural Network for COVID-19 Detection from Chest Radiographic Images. In. 2021 IEEE International Conference on Bioinformatics and Biomedicine (BIBM); 2021; Houston, TX, USA. pp. 1703–1710. 10.1109/bibm52615.2021.9669767 PMC9007173 35425662

[35] Ferreira JR, Cardenas DAC, Moreno RA, Rebelo MdFdS, Krieger JE, Gutierrez MA. Multi-View Ensemble Convolutional Neural Network to Improve Classification of Pneumonia in Low Contrast Chest X-Ray Images. In 2020 42nd Annual International Conference of the IEEE Engineering in Medicine & Biology Society (EMBC); 2020; Montreal, QC, Canada. pp. 1238–1241. 10.1109/EMBC44109.2020.9176517 33018211

[36] Anjum T, Chowdhury TE, Sakib S, Kibria S. Performance Analysis of Convolutional Neural Network Architectures for the Identification of COVID-19 from Chest X-ray Images. In 2022 IEEE 12th Annual Computing and Communication, Workshop, Conference. (CCWC); 2022; Las Vegas, NV, USA. pp. 446–452.

[37] Lafraxo S, Ansari M. CoviNet: Automated COVID-19 Detection from X-rays using Deep Learning Techniques. In 2020 6th IEEE Congress on Information Science and Technology (CiSt); 2020; Agadir - Essaouira, Morocco.

[38] Li J, Zhang D, Liu Q, Bu R, Wei Q, COVID-GATNet:. A Deep Learning Framework for Screening of COVID-19 from Chest X-Ray Images. In 2020 IEEE 6th International Conference on Computer and Communications (ICCC); 2020; Chengdu, China. pp. 1897–1902.

[39] Naveen P, Diwan B. Pre-trained VGG-16 with CNN Architecture to classify X-Rays images into Normal or Pneumonia. In 2021 International Conference on Emerging Smart Computing and Informatics (ESCI); 2021; Pune, India.

[40] Khan MA, Azhar M, Ibrar K, Alqahtani A, Alsubai S, Binbusayyis A et al. COVID-19 Classification from Chest X-Ray Images: A Framework of Deep Explainable Artificial Intelligence. Computational Intelligence and Neuroscience. 2022; 2022. 10.1155/2022/4254631 PMC9284325 35845911

[41] Ukwuoma CC, Qin Z, Heyat MBB, Akhtar F, Smahi A, Jackson JK et al. Automated Lung-Related pneumonia and COVID-19 detection based on novel feature extraction framework and vision transformer approaches using chest X-ray images. Bioengineering 2022;9(11). 10.3390/bioengineering9110709 PMC9687434 36421110

[42] Qi X, Brown LG, Foran DJ, Nosher J, Hacihaliloglu I. Chest X-ray image phase features for improved diagnosis of COVID-19 using convolutional neural network. Int J Comput Assist Radiol Surg. 2021;16(2):197–206. 33420641 10.1007/s11548-020-02305-w PMC7794081

[43] Sharifrazi D, Alizadehsani R, Roshanzamir M, Joloudari JH, Shoeibi A, Jafari M et al. Fusion of Convolution neural network, support vector machine and Sobel filter for accurate detection of COVID-19 patients using X-ray images. Biomed Signal Process Control. 2021;68. 10.1016/j.bspc.2021.102622 PMC8026268 33846685

[44] Sanghvi HA, Patel RH, Agarwal A, Gupta S, Sawhney V, Pandya AS. A deep learning approach for classification of COVID and pneumonia using DenseNet-201. Int J Imaging Syst Technol. 2022. 10.1002/ima.22812 PMC9537800 36249091

[45] Young An J, Seo H, Kim YG, Lee KE, Kim S, Kong HJ. Codeless deep learning of COVID-19 chest X-Ray image dataset with KNIME analytics platform. Healthc Inf Res. 2021;27(1):82–91. 10.4258/hir.2021.27.1.82 PMC7921566 33611880

[46] Babukarthik RG, Chandramohan D, Tripathi D, Kumar M, Sambasivam G. COVID-19 identification in chest X-ray images using intelligent multi-level classification scenario. Comput Electr Eng. 2022;104. 10.1016/j.compeleceng.2022.108405 PMC9510091 36187137

[47] Irmak E. Implementation of convolutional neural network approach for COVID-19 disease detection. Physiol Genomics. 2020;52(12):590–601. 33094700 10.1152/physiolgenomics.00084.2020 PMC7774002

[48] Ayan E, Karabulut B, Ünver M. Diagnosis of pediatric pneumonia with ensemble of deep convolutional neural networks in chest X-Ray images. Arab J Sci Eng. 2022;47(2):2123–39. 34540526 10.1007/s13369-021-06127-z PMC8435166

[49] Ali M, SM A, AU M, MMF A, ASC A, OSA A, DLTDI A, AI A. Pneumonia detection using chest radiographs with novel EfficientNetV2L model. IEEE Access. 2024 March;12:34691–707.

[50] Althenayan AS, AlSalamah SA, Aly S, Nouh T, Mahboub B, Salameh L, Alkubeyyer M, Mirza A. COVID-19 hierarchical classification using a deep learning Multi-Modal. Sens MDPI. 2024;24(8). 10.3390/s24082641 PMC11053684 38676257

[51] Okolo GI, Katsigiannis S, Ramzan N. IEViT: an enhanced vision transformer architecture for chest X-ray image classification. Comput Methods Programs Biomed. 2022;226. 10.1016/j.cmpb.2022.107141 36162246

[52] Murugan S, Venkatesan C, Sumithra MG, Gao XZ, Elakkiya B, Akila M, et al. DEMNET: A deep learning model for early diagnosis of alzheimer diseases and dementia from MR images. IEEE Access. 2021;9:90319–29.

[53] Tomassini S, Falcionelli N, Sernani P, Müller H, Dragoni AF. An End-to-End 3D ConvLSTM-based Framework for Early Diagnosis of Alzheimer’s Disease from Full-Resolution Whole-Brain sMRI Scans. In 2021 IEEE 34th International Symposium on Computer-Based Medical Systems (CBMS); 2021; Aveiro, Portugal. pp. 2–5.

[54] Yagis E, Citi L, Diciotti S, Marzi C, Atnafu SW, Herrera AGSD. 3D Convolutional Neural Networks for Diagnosis of Alzheimer’s Disease via Structural MRI. In 2020 IEEE 33rd International Symposium on Computer-Based Medical Systems (CBMS); 2020; Rochester, MN, USA.

[55] Jang J, Hwang D. M3T: three-dimensional Medical image classifier using Multi-plane and Multi-slice Transformer. In 2022 IEEE/CVF Conference on Computer Vision and Pattern Recognition (CVPR); 2022; New Orleans, LA, USA. pp. 20686–20697.

[56] Sahumbaiev I, Popov A, Ramírez J, Górriz JM, Ortiz A. 3D-CNN HadNet classification of MRI for Alzheimer’s Disease diagnosis. In 2018 IEEE Nuclear Science Symposium and Medical Imaging Conference Proceedings (NSS/MIC); 2018; Sydney, NSW, Australia.

[57] Cui R, Liu M, Li G. Longitudinal analysis for Alzheimer’s disease diagnosis using RNN. In 2018 IEEE 15th International Symposium on Biomedical Imaging (ISBI 2018); 2018; Washington, DC, USA. pp. 1–10.

[58] Puspaningrum EY, Wahid RR, Amaliyah RP, Nisa C. Alzheimer’s disease stage classification using deep convolutional neural networks on oversampled imbalance data. 2020 6th information technology international seminar. Surabaya, Indonesia: ITIS); 2020.

[59] Hussain E, Hasan M, Hassan SZ, Azmi TH, Rahman MA, Parvez MZ. Deep Learning Based Binary Classification for Alzheimer’s Disease Detection using Brain MRI Images. In 2020 15th IEEE Conference on Industrial Electronics and Applications (ICIEA); 2020; Kristiansand, Norway. pp. 1115–1120.

[60] Kushol R, Masoumzadeh A, Huo D, Kalra S, Yang YH, Addformer. Alzheimer’s Disease Detection from Structural Mri Using Fusion Transformer. In 2022 IEEE 19th International Symposium on Biomedical Imaging (ISBI); 2022; Kolkata, India.

[61] Menikdiwela M, Nguyen C, Shaw M. Deep Learning on Brain Cortical Thickness Data for Disease Classification. In 2018 Digital Image Computing: Techniques and Applications (DICTA); 2018; Canberra, ACT, Australia. pp. 1–5.

[62] Illakiya T, RK,SMV,MR,UA. AHANet. Adaptive Hybrid Attention Network for Alzheimer’s Disease Classification Using Brain Magnetic Resonance Imaging. Bioengineering, MDPI. 2023 June;10(6). 10.3390/bioengineering10060714 PMC10294993 37370645

[63] Ramzan F, Khan MUG, Rehmat A, Iqbal S, Saba T, Rehman A et al. A deep learning approach for automated diagnosis and Multi-Class classification of Alzheimer’s disease stages using Resting-State fMRI and residual neural networks. J Med Syst. 2020;44(2). 10.1007/s10916-019-1475-2 31853655

[64] Wegmayr V, Aitharaju S, Buhmann J. Classification of brain MRI with big data and deep 3D convolutional neural networks. In Progress in Biomedical Optics and Imaging - Proceedings of SPIE.

[65] Fareed MMS, Zikria S, Ahmed G, Mui-Zzud-Din, Mahmood S, Aslam M, et al. ADD-Net: an effective deep learning model for early detection of alzheimer disease in MRI scans. IEEE ACCESS. 2022;10:96930–51.

[66] Shahamat H, Abadeh MS. Brain MRI analysis using a deep learning based evolutionary approach. Neural Netw. 2020;126:218–34. 32259762 10.1016/j.neunet.2020.03.017

[67] Kim E, Kim S, Seo M, Yoon S. XProtoNet: Diagnosis in Chest Radiography with Global and Local Explanations. In 2021 IEEE/CVF Conference on Computer Vision and Pattern Recognition (CVPR); 2021; Nashville, TN, USA. pp. 15719–15728.

[68] Wang X, Peng Y, Lu L, Lu Z, Bagheri M, Summers RM, Recognition P. ChestX-Ray8: Hospital-Scale Chest X-Ray Database and Benchmarks on Weakly-Supervised Classification and Localization of Common Thorax Diseases. In (CVPR); 2017; Honolulu, HI, USA. pp. 2097–2106.

[69] Wang K, Zhang X, Huang S. KGZNet:Knowledge-Guided Deep Zoom Neural Networks for Thoracic Disease Classification. In. 2019 IEEE International Conference on Bioinformatics and Biomedicine (BIBM); 2019; San Diego, CA, USA.

[70] Teixeira V, Braz L, Pedrini H, Dias Z, DuaLAnet. Dual Lesion Attention Network for Thoracic Disease Classification in Chest X-Rays. In 2020 International Conference on Systems, Signals and Image Processing (IWSSIP); 2020; Niteroi, Brazil.

[71] Jaipurkar SS, Jie W, Zeng Z, Gee TS, Veeravalli B, Chua M. Automated Classification Using End-to-End Deep Learning. In. 2018 40th Annual International Conference of the IEEE Engineering in Medicine and Biology Society (EMBC); 2018; Honolulu, HI, USA. pp. 706–709. 10.1109/EMBC.2018.8512356 30440494

[72] Hossain I, ZM A, AK M, HJ A, HA M, HT M. ThoraX-PriorNet: A novel attention-based architecture using anatomical prior probability maps for thoracic disease classification. IEEE Access. 2024 January;12:3256–73.

[73] Bhanothu Y, Kamalakannan A, Rajamanickam G, Systems C. Detection and Classification of Brain Tumor in MRI Images using Deep Convolutional Network. In (ICACCS); 2020; Coimbatore, India.

[74] Dipu NM, Shohan SA, Salam KMA. Deep Learning Based Brain Tumor Detection and Classification. In 2021 International Conference on Intelligent Technologies (CONIT); 2021; Hubli, India.

[75] El Kader IA, Xu G, Shuai Z, Saminu S, Javaid I, Ahmad IS. Differential deep convolutional neural network model for brain tumor classification. Brain Sci. 2021;11(3). 10.3390/brainsci11030352 PMC8001442 33801994

[76] Papadomanolakis TN, Sergaki ES, Polydorou AA, Krasoudakis AG, Makris-Tsalikis GN, Polydorou AA et al. Tumor diagnosis against other brain diseases using T2 MRI brain images and CNN binary classifier and DWT. Brain Sci. 2023;13(2). 10.3390/brainsci13020348 PMC9954603 36831891

[77] Rastogi D, Johri P, Tiwari V, Elngar A. Multi-class classification of brain tumour magnetic resonance images using multi-branch network with inception block and five-fold cross validation deep learning framework. Biomedical Signal Processing and Control; 2024.

[78] Dikande Simo AM, Tchagna Kouanou A, Monthe V, Kameni Nana M, Moffo Lonla B. Introducing a deep learning method for brain tumor classification using MRI data towards better performance. Inf Med Unlocked. 2023;44.

[79] Mzoughi H, Njeh I, Wali A, Slima MB, BenHamida A, Mhiri C, et al. Deep Multi-Scale 3D convolutional neural network (CNN) for MRI gliomas brain tumor classification. J Digit Imaging. 2020;33:903–15. 32440926 10.1007/s10278-020-00347-9 PMC7522155

[80] Zhao X, Shen X, Wan W, Lu Y, Hu S, Xiao R, et al. Automatic thyroid ultrasound image classification using feature fusion network. IEEE Access. 2022;10:27917–24.

[81] Zhang S, Du H, Jin Z, Zhu Y, Zhang Y, Xie F, et al. A novel interpretable Computer-Aided diagnosis system of thyroid nodules on ultrasound based on clinical experience. IEEE Access. 2020;8:53223–31.

[82] Manh VT, Zhou J, Jia X, Lin Z, Xu W, Mei Z, et al. Multi-Attribute attention network for interpretable diagnosis of thyroid nodules in ultrasound images. IEEE Trans Ultrason Ferroelectr Freq Control. 2022;69(9):2611–20. 35820014 10.1109/TUFFC.2022.3190012

[83] Yang J, Shi X, Wang B, Qiu W, Tian G, Wang X et al. Ultrasound Image Classification of Thyroid Nodules Based on Deep Learning. Frontiers, Sec. Cancer Imaging and Image-directed. 2022;12. 10.3389/fonc.2022.905955 PMC9335944 35912199

[84] Zhang B, Vakanski A, Xian M. Bi-Rads-Net: An Explainable Multitask Learning Approach for Cancer Diagnosis in Breast Ultrasound Images. In 2021 IEEE 31st International Workshop on Machine Learning for Signal Processing (MLSP); 2021; Gold Coast, Australia. 10.1109/mlsp52302.2021.9596314 PMC9063460 35509454

[85] Kim J, Kim HJ, Kim C, Lee JW, Kim KW, Park YM et al. Weakly-supervised deep learning for ultrasound diagnosis of breast cancer. Sci Rep. 2021;11(1). 10.1038/s41598-021-03806-7 PMC8692405 34934144

[86] Ma H, Tian R, Li H, Sun H, Lu H, Lu G et al. Fus2Net: a novel convolutional neural network for classification of benign and malignant breast tumor in ultrasound images. Biomed Eng Online. 2021;20(1). 10.1186/s12938-021-00950-z PMC8600702 34794443

[87] Raza A, Ullah N, Khan JA, Assam M, Guzzo A, Aljuaid H. DeepBreastCancerNet: A novel deep learning model for breast Cancer detection using ultrasound images. Appl Scienced. 2023;13(4).

[88] Zhuang Z, Yang Z, Raj ANJ, Wei C, Jin P, Zhuang S. Breast ultrasound tumor image classification using image decomposition and fusion based on adaptive multi-model Spatial feature fusion. Comput Methods Programs Biomed. 2021; 208. 10.1016/j.cmpb.2021.106221 34144251

[89] Sahu A, Das PK, Meher S. An efficient deep learning scheme to detect breast cancer using mammogram and ultrasound breast images. Biomed Signal Process Control. 2023;87.

[90] Sirjani N, Oghli MG, Tarzamni MK, Gity M, Shabanzadeh A, Ghaderi P, et al. A novel deep learning model for breast lesion classification using ultrasound images: A multicenter data evaluation. Phys Med. 2023. p. 107. 10.1016/j.ejmp.2023.102560 36878133

[91] Latha M, Santhosh Kumar P, Roopa Chandrika R, Mahesh TR, Vinoth Kumar V, Guluwadi S. Revolutionizing breast ultrasound diagnostics with EfficientNet-B7 and explainable AI. BMC Med Imaging. 2024;24. 10.1186/s12880-024-01404-3 PMC11367906 39223507

[92] Jiménez-Sánchez A, Kazi A, Albarqouni S, Kirchhoff C, Biberthaler P, Navab N, et al. Precise proximal femur fracture classification for interactive training and surgical planning. Int J Comput Assist Radiol Surg. 2020;15(5):847–57. 32335786 10.1007/s11548-020-02150-x

[93] Yadav DP, Sharma A, Athithan S, Bhola A, Sharma B, Dhaou IB. Hybrid SFNet model for bone fracture detection and classification using ML/DL. Sensors. 2022;22(15). 10.3390/s22155823 PMC9371081 35957380

[94] Xu T, Yuan Z. Convolution neural network with coordinate attention for the automatic detection of pulmonary tuberculosis images on chest X-Rays. IEEE Access. 2022;10:86710–7.

[95] Liu C, Cao Y, Alcantara M, Liu B, Brunette M, Peinado J et al. TX-CNN: Detecting tuberculosis in chest X-ray images using convolutional neural network. In. 2017 IEEE International Conference on Image Processing (ICIP); 2017; Beijing, China.

[96] Sharma V, Nillmani, Gupta SK, Shukla KK. Deep learning models for tuberculosis detection and infected region visualization in chest X-ray images. Intell Med. 2024;4(2):104–13.

[97] Trivizakis E, Manikis GC, Nikiforaki K, Drevelegas K, Constantinides M, Drevelegas A, et al. Extending 2D convolutional neural networks to 3D for advancing deep learning Cancer classification with application to MRI liver tumor differentiation. IEEE J Biomedical Health Inf. 2019;23(3):923–30. 10.1109/JBHI.2018.2886276 30561355

[98] Zhao T, Zeng Z, Li T, Tao W, Yu X, Feng T et al. USC-ENet: a high-efficiency model for the diagnosis of liver tumors combining B-mode ultrasound and clinical data. Health Inform Sci Syst. 2023;11(1). 10.1007/s13755-023-00217-y PMC10025174 36950106

[99] Joo Y, Park H, Lee O, Yoon C, Choi MH, Choi C. Classification of liver fibrosis from heterogeneous ultrasound image. IEEE Access. 2023;11:9920–30.

[100] Huang Y, Zeng Y, Bin G, Ding Q, Wu S, Tai D et al. Evaluation of hepatic fibrosis using ultrasound backscattered. Diagnostics (MDPI). 2022;12(11). 10.3390/diagnostics12112833 PMC9689172 36428892

[101] Aghdam MA, Sharifi A, Pedram MM. Diagnosis of autism spectrum disorders in young children based on Resting-State functional magnetic resonance imaging data using convolutional neural networks. J Digit Imaging. 2019;32(6):899–918. 30963340 10.1007/s10278-019-00196-1 PMC6841914

[102] Yang H, Wu H, Kong L, Luo W, Xie Q, Pan J, Quan W, Hu L, Li D, Wu X, Liang H, Qin P. Precise detection of awareness in disorders of consciousness using deep learning framework. NeuroImage. 2024 March; 290. 10.1016/j.neuroimage.2024.120580 38508294

[103] Fernández IS, Yang E, Calvachi P, Amengual-Gual M, Wu JY, Krueger D et al. Deep learning in rare disease. Detection of tubers in tuberous sclerosis complex. PLoS ONE. 2020;15(4). 10.1371/journal.pone.0232376 PMC7190137 32348367

[104] Salehi E, Khanbare S, Yousefi H, Sharpasand H, Sheyjani OS. Deep Convolutional Neural Networks for Automated Diagnosis of Disc Herniation on Axial MRI. In 2019 Scientific Meeting on Electrical-Electronics & Biomedical Engineering and Computer Science (EBBT); 2019; Istanbul, Turkey.

[105] Eisenstat J, Wagner MW, Vidarsson L, Ertl-Wagne B, Sussman D. Fet-Net algorithm for automatic detection of fetal orientation in fetal MRI. Bioengineering. 2023;10(2). 10.3390/bioengineering10020140 PMC9952178 36829634

[106] Pollack BL, Batmanghelich K, Cai SS, Gordon E, Wallace S, Catania R et al. Deep Learning Prediction of Voxel-Level Liver Stiffness in Patients with Nonalcoholic Fatty Liver Disease. Radiology: Artificial Intelligence. 2021;3(6). 10.1148/ryai.2021200274 PMC8637225 34870213

[107] Zhu Y, Wang L, Liu M, Qian C, Yousuf A, Oto A, et al. MRI-based prostate cancer detection with high-level representation and hierarchical classification. Med Phys. 2017;44(3):1028–39. 28107548 10.1002/mp.12116 PMC5540150

[108] Solatidehkordi Z, Zualkernan I. Survey on recent trends in medical image classification using Semi-Supervised learning. Appl Sci. 2022;12(23).

[109] Yadav SS, Jadhav SM. Deep convolutional neural network based medical image classifcation for disease diagnosis. J Big Data. 2019;113(6).

[110] Maharana K, Mondal S, Nemade B. A review: Data pre-processing and data augmentation techniques. In Global Transitions Proceedings; 2022. pp. 91–99.

[111] Shatnawi AM, Al-Bdour G, Al-Qurran RL, Al-Ayyoub M. A Comparative Study of Open Source Deep Learning Frameworks. In International Conference on Information and Communication Systems; 2018; Jordan.

[112] Nguyen G, Dlugolinsky S, Bobák M, Tran V, García ÁL, Heredia I, et al. Machine learning and deep learning frameworks and libraries for large-scale data mining: a survey. Artif Intell Rev. 2019;52:77–124.

[113] Goldsborough P. A Tour of TensorFlow. arXiv. 2016.

[114] Sarker IH. Deep learning: A comprehensive overview on techniques, taxonomy, applications and research directions. SN Comput Sci. 2021;2(6):420. 34426802 10.1007/s42979-021-00815-1 PMC8372231

[115] Singaravel S, Suykens JAK, Geyer P. Deep-learning neural-network architectures and methods: using component-based models in building-design energy prediction. Adv Eng Inform. 2018;38(4):81–90.

[116] Lundervold AS, Lundervold A. An overview of deep learning in medical imaging focusing on MRI. Z Med Phys. 2019;29(2):102–27. 30553609 10.1016/j.zemedi.2018.11.002

[117] Li X, Xiong H, Li X, Wu X, Zhang X, Liu J, et al. Interpretable deep learning: interpretation, interpretability, trustworthiness, and beyond. Knowl Inf Syst. 2022;64(12):3197–234.

[118] Dong G, Ma Y, Basu A. Feature-Guided CNN for denoising images from portable ultrasound devices. IEEE Access. 2021;9:28272–81.

[119] Dong G, Basu A. Medical image denosing via explainable AI feature preserving loss. arXiv: Electrical Engineering and Systems Science; 2023.

[120] Dang K, Vo T, Ngo L, Ha H. A deep learning framework integrating MRI image preprocessing methods for brain tumor segmentation and classification. IBRO Neurosci Rep. 2022;13:523–32. 36590099 10.1016/j.ibneur.2022.10.014 PMC9795279

[121] Kaur A, Dong G, Basu A. GradXcepUNet: Explainable AI Based Medical Image Segmentation. In Smart Multimedia, Marseille. France. pp. 174–188.

